# Predictive Values of Early Suppression of Tumorigenicity 2 for Acute GVHD and Transplant-related Complications after Allogeneic Stem Cell Transplantation: Prospective Observational Study

**DOI:** 10.4274/tjh.galenos.2019.2019.0139

**Published:** 2020-02-20

**Authors:** Ayako Matsumura, Takuya Miyazaki, Takayoshi Tachibana, Taiki Ando, Megumi Koyama, Satoshi Koyama, Yoshimi Ishii, Hiroyuki Takahashi, Yuki Nakajima, Ayumi Numata, Wataru Yamamoto, Kenji Motohashi, Maki Hagihara, Kenji Matsumoto, Shin Fujisawa, Hideaki Nakajima

**Affiliations:** 1 Yokohama City University Graduate School of Medicine, Department of Stem Cell and Immune Regulation, Kanagawa, Japan; 2 Yokohama City University Medical Center, Department of Hematology, Kanagawa, Japan

**Keywords:** Suppression of tumorigenicity 2, Graft-versus-host disease, Biomarker, Hematopoietic stem cell transplantation

## Abstract

**Objective:**

A soluble form of suppression of tumorigenicity 2 (sST2) has emerged as a biomarker for acute graft-versus-host disease (GVHD) and non-relapse mortality (NRM). We prospectively monitored sST2 levels during the early phase of hematopoietic stem cell transplantation (HSCT) and evaluated the clinical association with transplant-related complications including acute GVHD.

**Materials and Methods:**

Thirty-two adult Japanese patients who received a first allogeneic HSCT were enrolled in this study. Levels of sST2 were measured at fixed time points (pre-conditioning, day 0, day 14, day 21, and day 28).

**Results:**

The median age was 50.5 years (range=16-66). With a median follow-up of 21.5 months (range=0.9-35.4), 9 patients developed grade II-IV acute GVHD. Median sST2 levels on the day of HSCT were higher than baseline and reached the maximum value (92.7 ng/mL; range=0-419.7) on day 21 after HSCT. The optimal cut-off value of sST2 on day 14 for predicting grade II-IV acute GVHD was determined as 100 ng/mL by ROC analysis. The cumulative incidence of acute GVHD was 56.7% and 16.5% in the high- and low-sST2 groups, respectively (p<0.01). Multivariate analyses showed that high sST2 levels at day 14 were associated with a higher incidence of acute GVHD (hazard ratio=9.35, 95% confidence interval=2.92-30.0, p<0.01). The cumulative incidence of NRM was increased in the high-sST2 group (33% vs 0%, p<0.01), but all the patients died of non-GVHD complications. Among 6 patients in the high-sST2 group without grade II-IV GVHD, 5 patients developed veno-occlusive disease (VOD) and one also had thrombotic microangiopathy (TMA).

**Conclusion:**

The early assessment of sST2 after HSCT yielded predictive values for the onset of acute GVHD and other transplant-related complications including VOD and TMA.

## Abstract (tr)

**Amaç:**

Akut graft-konakçı hastalığı (GVHD) ve relaps dışı mortalite (NRM) için bir biyobelirteç olarak, tümör oluşumu 2’nin baskılanmasının (sST2) çözünür bir formu ortaya konulmuştur. Hematopoietik kök hücre transplantasyonunun (HSCT) erken safhasında sST2 düzeylerini prospektif olarak izledik ve akut GVHD dahil transplantla ilişkili komplikasyonlarla klinik ilişkisini değerlendirdik.

**Gereç ve Yöntemler:**

Bu çalışmaya ilk allojenik HSCT’si olan 32 yetişkin Japon hasta alındı. sST2 seviyeleri sabit zaman noktalarında (hazırlık rejimi öncesi, 0. gün, 14. gün, 21. gün ve 28. gün) ölçüldü.

**Bulgular:**

Ortanca yaş 50,5 idi (aralık=16-66). Ortalama 21,5 aylık takip süresi (aralık= 0,9-35,4) ile 9 hastada Grade II-IV akut GVHD gelişti. Nakil gününde ortanca sST2 seviyeleri bazal değerlerden daha yüksekti ve HSCT’den sonraki 21. günde maksimum değere (92,7 ng/mL; aralık=0-419,7) ulaştı. Grade II-IV akut GVHD’yi tahmin etmek için 14. günde sST2’nin optimal eşik değeri, ROC analizi ile 100 ng/mL olarak belirlenmiştir. Akut GVHD’nin kümülatif insidansı yüksek ve düşük sST2 gruplarında sırasıyla %56,7 ve %16,5 idi (p<0,01). Çok değişkenli analizler 14. günde yüksek sST2 düzeylerinin daha yüksek akut GVHD insidansı ile ilişkili olduğunu göstermiştir (tehlike oranı=9, 35, %95 güven aralığı=2,92-30,0, p<0,01). NRM’nin kümülatif insidansı yüksek sST2 grubunda artmıştır (%33’e karşı %0, p<0,01), ancak tüm hastalar GVHD dışı komplikasyonlardan öldü. Yüksek sST2 grubunda grade II-IV GVHD olmayan 6 hastadan 5’inde veno-oklüzif hastalık (VOD) gelişti ve birinde trombotik mikroanjiyopati (TMA) vardı.

**Sonuç:**

HSCT sonrası sST2’nin erken değerlendirmesi, akut GVHD’nin başlangıcı ve VOD ve TMA dahil transplantla ilişkili diğer komplikasyonlar için prediktif değerler vermiştir.

## Introduction

Acute graft-versus-host disease (GVHD) is one of the major complications after allogeneic hematopoietic stem cell transplantation (HSCT) and remains the leading cause of non-relapse mortality (NRM) [1,2]. Identifying useful biomarkers for predicting onset or severity of acute GVHD at the early phase of HSCT may help in the development of a more individualized treatment strategy for GVHD. Recent studies show several plasma biomarkers that correlate with acute GVHD: suppression of tumorigenicity 2 (ST2) [3,4], interleukin (IL)-2 receptor α [5], tumor necrosis factor receptor 1 (TNFR1) [5], hepatocyte growth factor [5], IL-8 [5], and IL-6 [6] for systemic GVHD; elafin for skin GVHD [7]; and regenerating islet-derived 3-α [8] and T-cell immunoglobulin mucin-3 for gastrointestinal GVHD [8,9,10,11,12]. Among these biomarkers, ST2 has emerged as a promising biomarker for onset or steroid-resistant acute GVHD and NRM [3,4,12,13,14]. In addition, recent studies have demonstrated that high ST2 levels are associated with the development of thrombotic microangiopathy (TMA) and veno-occlusive disease (VOD) after HSCT [15,16].

ST2 is a membrane receptor expressed on several immune cell types that belong to the IL-1 receptor family. ST2 induces T helper type 2 (Th2) and Treg immune responses, which play important roles in GVHD [17]. A soluble form of ST2 (sST2) is a decoy receptor for IL-33, which blocks the IL-33/ST2 pathway and drives Th2 cells toward a Th1 cell phenotype, and it is thought to be important in the pathophysiology of GVHD [18,19]. In murine models, the ST2/IL-33 axis has been reported as a potential therapeutic target for GVHD [20,21].

Despite the accumulating evidence that higher sST2 levels are associated with increased GVHD risk and subsequent mortality, a clinical role of sST2 during the early phase of transplantation has not been fully elucidated. We performed a multicenter, prospective, observational study monitoring the serial changes in sST2 levels before and early after HSCT with the aim of determining the diagnostic and prognostic values for acute GVHD, other transplant-related complications, and mortality.

## Materials and Methods

### Patients and Transplant Procedure

Patients who received first allogeneic HSCT for hematological diseases were enrolled consecutively between February 2014 and July 2015 at Yokohama City University Hospital or Yokohama City University Medical Center. The selection of donor source and conditioning regimen was based on patients’ hematological diagnosis, donor availability, and patients’ clinical status.

Conditioning regimens were classified into myeloablative conditioning (MAC) and reduced-intensity conditioning (RIC) according to the definitions previously reported [22]. The pre-transplant risk category was defined as standard or high according to the diagnosis and the disease stage at the time of transplantation, as described previously [23].

Written informed consent was obtained from all the patients enrolled in this study before the start of the trial. This study was approved by the institutional review boards of our university hospital and medical center and was conducted in accordance with the Declaration of Helsinki. All authors vouched for the accuracy and completeness of the reported data, analyses, and adherence to the study protocol.

### Peripheral Blood Samples

Samples were collected prospectively before conditioning, on the day of HSCT (day 0), and on days 14, 21, and 28 after HSCT. Serum sST2 and IL-33 levels were measured by ELISA (Human ST2/IL-1 R4 ELISA Kit and Human IL-33 ELISA Kit; R&D Systems, Minneapolis, MN, USA). Absorbance was measured using a microplate reader (Powerscan HT, DS Pharma Biomedical, Osaka, Japan).

### Transplant-related Complications

Acute GVHD was diagnosed clinically with histological confirmation when available. The classification of acute GVHD was based on the diagnostic criteria of the 1994 Consensus Conference on Acute GVHD Grading [24]. GVHD prophylaxis consisted of cyclosporine or tacrolimus with short-term methotrexate. Anti-thymocyte globulin was administered for HLA-serological mismatched transplant based on each institution’s criteria.

Other transplant-related complications were diagnosed as follows: TMA was diagnosed according to the Blood and Marrow Clinical Trials Network and European Group for Blood and Marrow Transplantation guidelines [25,26]. VOD was diagnosed based on the Baltimore and Seattle criteria [27,28].

### Statistical Analysis

Fisher’s exact test and the Mann-Whitney U test were used to assess the categorical and continuous variables, respectively. The receiver operating characteristics (ROC) curve from logistic regression models with the area under the curve (AUC) was used to present the correlation between sST2 and other biomarkers. The Pearson test was used to determine the correlation between sST2 and other biomarkers. The Kaplan-Meier method was used to assess overall survival (OS) using the log-rank test. For multivariate analysis, the Cox proportional hazards method was used to assess the OS. The Gray test and Fine-Gray test were used to assess the cumulative incidence of GVHD and NRM. The competing risks were GVHD and death prior to GVHD. Values of p<0.05 were considered statistically significant. All analyses were performed using EZR version 1.36 statistical software, which is a graphical user interface for R version 3.4.1 [29].

## Results

### Patient Characteristics

The clinical characteristics of 32 patients are summarized in Table 1. The transplant procedure was deemed heterogeneous based on the donor source, conditioning regimen, and GVHD prophylaxis.

### Expression Patterns of sST2 Following Transplantation

**Figure 1:**
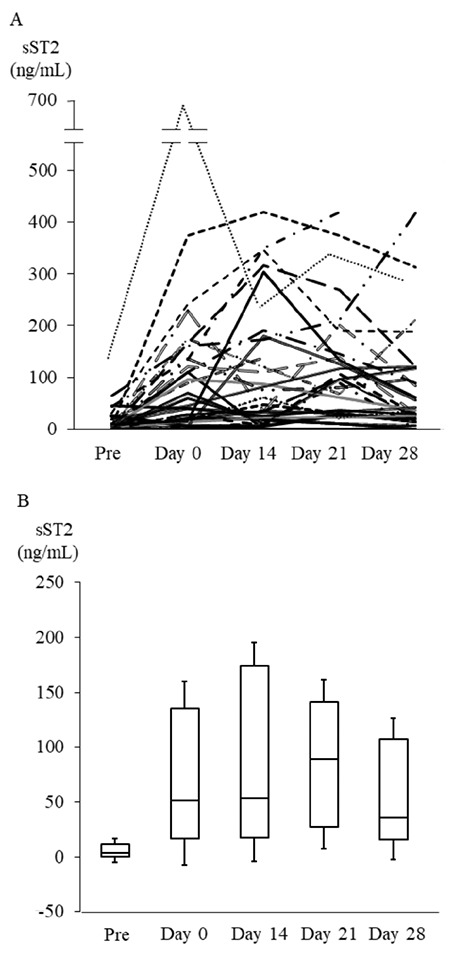
Expression patterns of sST2 following transplantation. A) Expression patterns of sST2 before conditioning (Pre) and on days 0, 14, 21, and 28 after transplantation in individual patients. B) Expression patterns of median sST2 values before conditioning (Pre) and on days 0, 14, 21, and 28 after transplantation. Box plots indicate medians, interquartiles, and ranges of sST2 levels. sST2: Suppression of tumorigenicity 2.

First, we evaluated the expression patterns of sST2 by serial sampling from individuals at a fixed time point before and early after HSCT. Different sST2 expression patterns were observed in each individual after the conditioning therapy (Figure 1A). Compared with median sST2 levels before conditioning (median=25.9 ng/mL; range=0-42.7 ng/mL), sST2 levels on day 0 were remarkably elevated in most of the patients (median=51.4 ng/mL; range=0-227.9 ng/mL). The sST2 levels reached the maximum value on day 21 after HSCT (median=92.7 ng/mL; range=0-419.7 ng/mL) (Figure 1B).

### Effects of Conditioning Damages and Inflammatory Conditions on sST2 Levels

**Figure 2:**
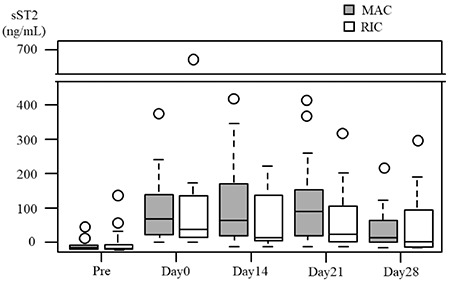
Comparison of sST2 expression patterns based on conditioning intensity. Median sST2 levels in patients who received MAC and RIC following transplantation. Box plots indicate medians, interquartiles, and ranges of sST2 levels. RIC: Reduced-intensity conditioning, MAC: Myeloablative conditioning, sST2: Suppression of tumorigenicity 2.

As sST2 expression levels may depend on conditioning intensity [4], the sST2 levels in patients who underwent MAC and RIC were compared. The sST2 levels in patients who underwent MAC were higher than those in patients who underwent RIC on days 0, 14, 21, and 28 without statistical difference between the two groups (Figure 2). To determine whether sST2 was affected by various inflammatory conditions after HSCT, the correlations between sST2 and representative inflammatory markers, serum ferritin and C-reactive protein (CRP), were estimated (Table 2). sST2 levels were strongly correlated with ferritin and CRP levels in all samples (r=0.456 and 0.615, respectively). Specifically, sST2 was well correlated with CRP at days 0, 14, and 21 (r=0.717, 0.630, and 0.628, respectively). Furthermore, levels of serum IL-33, the ligand of ST2, were under the detection limits in most of the patients, resulting in no correlation of IL-33 with sST2 levels (data not shown).

### Association of sST2 Levels with Onset of Acute GVHD

**Figure 3:**
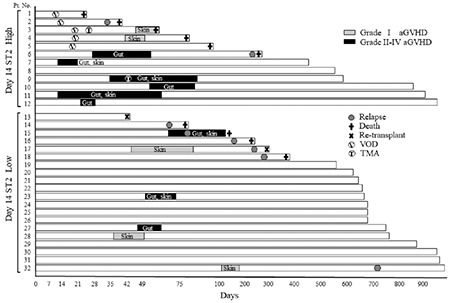
Clinical courses following HSCT in individual patients. aGVHD: Acute graft-versus-host disease, Pt. No.: Patient number, VOD: Veno-occlusive disease, TMA: Thrombotic microangiopathy, ST2: Suppression of tumorigenicity 2, HSCT: Hematopoietic stem cell transplantation.

The clinical courses of all patients are summarized in Figure 3. With a median follow-up of 21.5 months (range=0.9-35.4) after HSCT, 14 patients (43%) developed some grade of acute GVHD [median days to onset=39 days (range=9-84); median days to maximum grade=44 (range=15-94)]. The maximum grade of GVHD was grade I in five patients, grade II in three, and grade III in six; none developed grade IV. Nine patients had involvement of the gastrointestinal tract (stage 1 in two patients, stage 2 in two, stage 3 in four, and stage 4 in one), and ten patients had skin involvement (stage 1 in one patient, stage 2 in five, and stage 3 in four). None developed liver GVHD.

**Figure 4:**
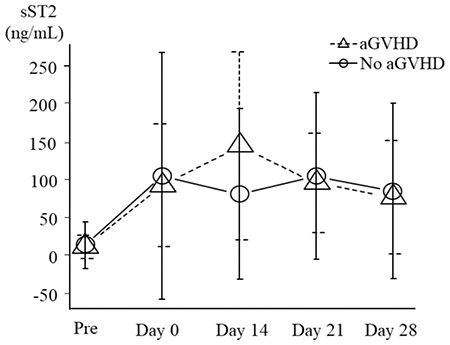
Comparison of sST2 expression patterns in patients with and without acute GVHD. Mean sST2 levels in patients who developed grade II-IV acute GVHD or not following transplantation. Bars indicate the mean ± SEM of sST2 levels. aGVHD: Acute graft-versus-host disease, sST2: Suppression of tumorigenicity 2, GVHD: Graft-versus-host disease.

**Figure 5:**
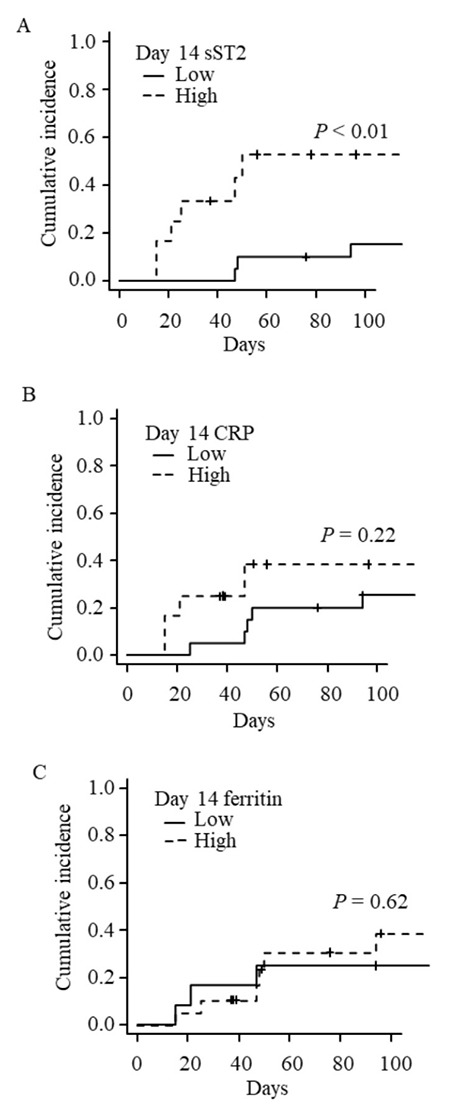
High sST2 on day 14 is correlated with subsequent GVHD development. A) Cumulative incidence of grade II-IV acute GVHD by day 100 in patients with high sST2 (>100 ng/mL) and low sST2 (≤100 ng/mL) levels on day 14 after transplantation. B) Cumulative incidence of grade II-IV acute GVHD by day 100 in patients with high-CRP (>6.0 mg/dL) and low-CRP (≤6.0 mg/dL) levels on day 14 after transplantation. C) Cumulative incidence of grade II-IV acute GVHD by day 100 in patients with high ferritin (>1700 mg/dL) and low ferritin (≤1700 mg/dL) levels on day 14 after transplantation. The CRP and ferritin cut-off values were calculated by ROC curve analysis for predicting the onset of acute GVHD (ROC curves are available in Figure S1). sST2: Suppression of tumorigenicity 2, CRP: C-reactive protein, GVHD: Graft-versus-host disease.

**Figure S1:**
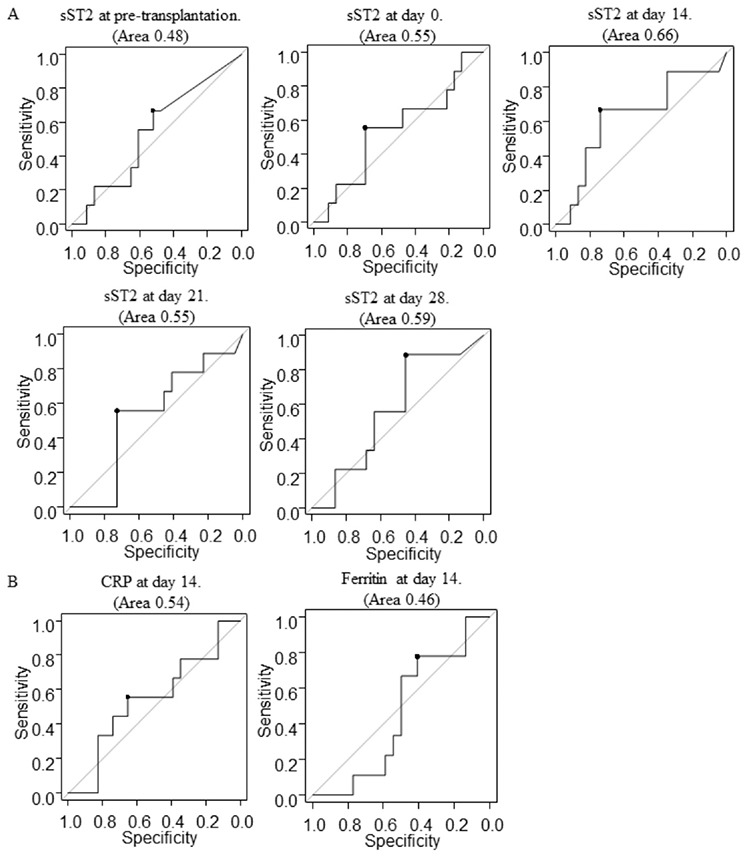
ROC curves of sST2, CRP, and ferritin for predicting the development of acute GVHD. A) ROC curves for sST2 with area under the curve (AUC) before conditioning and on days 0, 14, 21, and 28 after transplantation. B) ROC curves for CRP and ferritin with AUC on day 14. sST2: Suppression of tumorigenicity 2, CRP: C-reactive protein, GVHD: Graft-versus-host disease, AUC: Area under the curve, ROC: Receiver operating characteristics.

When comparing the sST2 levels in patients with and without GVHD, median sST2 levels on day 14 after HSCT were relatively higher in patients with GVHD, but with no significant difference (Figure 4). Based on ROC curve analyses for predicting the onset of acute GVHD, the sST2 levels on day 14 showed the best AUC (0.66), with 66.7% sensitivity and 73.9% specificity (Figure S1, A). The optimal cut-off point of sST2 was 100 ng/mL based on the ROC analysis. We focused on day 14 sST2 levels as the earliest time point for predicting acute GVHD. There was no significant difference in patient characteristics between the high-sST2 group (>100 ng/mL) and the low-sST2 group (≤100 ng/mL) (Table 1). The cumulative incidence of grades II-IV GVHD was significantly higher in the high-sST2 group (56.7%) than that in the low-sST2 group (16.5%) (p<0.01) (Figure 5A). Multivariate analyses showed that high sST2 was associated with higher incidence of acute GVHD (hazard ratio=9.35, 95% confidence interval=2.92-30.0, p<0.01) (Table 3). sST2 levels at other time points of days 0, 21, and 28 were not associated with acute GVHD (data not shown). Although post-transplant CRP and ferritin levels were well correlated with sST2 (Table 2), these markers did not influence the cumulative incidence of acute GVHD (cut-off values were calculated by ROC analysis=CRP 6.0 mg/dL and ferritin 1700 mg/dL, respectively) (Figure 5B and Figure S1, B). These data suggest that the day 14 sST2 levels are supportive findings for predicting the onset of acute GVHD.

**Figure 6:**
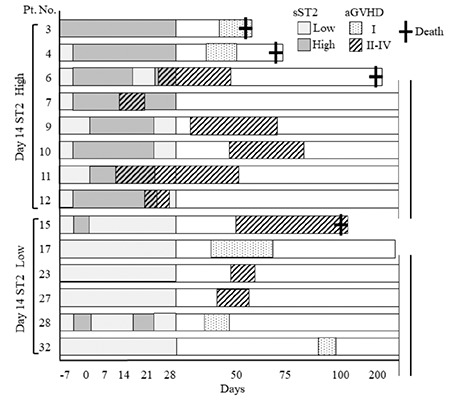
Association of early-phase sST2 levels with subsequent development of acute GVHD. Patient numbers correspond with those in Figure 3. Clinical course of 14 patients who developed grade I-IV acute GVHD. Dotted bars (grade I GVHD) or striped bars (grade II-IV GVHD) indicate the duration of acute GVHD clinical symptoms. Cut-off point of sST2 is 100 ng/mL. aGVHD: Acute graft-versus-host disease, Pt. No.: Patient number, GVHD: Graft-versus-host disease, sST2: Suppression of tumorigenicity 2.

Among the nine patients who had grade II-IV GVHD, six patients were part of the high-sST2 group (Figure 6). The patients in the high-sST2 group developed acute GVHD relatively early after HSCT (median onset of GVHD=22 days; range=9-50), and sST2 levels were elevated several days before the onset of acute GVHD. Four patients (cases 6, 7, 11, and 12) developed acute GVHD before day 28, and the sST2 levels were the highest just before the development of GVHD. On the other hand, low-sST2 patients did not develop GVHD in the early phase of HSCT (median onset of GVHD=48 days; range=42-55).

### Association of sST2 Levels with GVHD Severity and Target Organ Involvement

The association of day 14 sST2 levels with GVHD severity was examined, but the sST2 levels were not associated with the grade of GVHD (data not shown). The cumulative incidence of gastrointestinal GVHD was significantly increased in the high-sST2 group (50% vs. 15%, p=0.03). However, there was no significant association between sST2 levels and skin GVHD.

### Association of sST2 Levels with Other Transplant-related Complications

Finally, we evaluated the association of sST2 levels with other transplant-related complications and mortality (Figure 3). The 1-year NRM and OS after HSCT was 12.5% and 65.6%, respectively. The cumulative incidence of 1-year NRM was significantly increased in the high-sST2 group on day 14 (33% vs. 0%, p<0.01), while there was no significant difference in 1-year OS by univariate analysis (high sST2=50.0% vs. low sST2=84.2%; p=0.11). Severe non-GVHD complications were observed in 10 patients, including TMA (n=2), VOD (n=5), graft failure (n=1), engraftment syndrome (n=4), sepsis (n=3), and pneumonia (n=1), which often overlapped. Four patients in the high-sST2 group died of transplant-related complications including sepsis, graft failure, TMA, and pneumonia (cases 1, 3, 4, and 5) without developing grade II-IV GVHD. The two patients who had TMA (cases 3 and 9) and five who had VOD (cases 1-5) were included in the high-ST2 group. All patients who died had severe overlapping complications including VOD.

## Discussion

Previous studies have shown that ST2 may predict the onset of acute GVHD, but no detailed analysis regarding the serial monitoring of sST2 following HSCT has been reported thus far. In this study, we performed a prospective evaluation of sST2 expression patterns by serial monitoring before and early after HSCT and identified that early assessment of sST2 after HSCT can yield predictive values for the onset of acute GVHD in addition to other severe transplant-related complications, such as TMA and VOD.

Although high sST2 levels on day 28 are associated with an increased risk of acute GVHD in cord blood transplantation [2], the optimal timing to measure sST2 remains unclear. We conducted serial sST2 measurements at fixed time points during HSCT and found that high sST2 levels around day 14 had the most significant association for predicting GVHD development. The expression patterns of sST2 in individual patients showed that sST2 is relatively higher during exacerbation of acute GVHD. As shown in Figure 6, the sST2 levels in patients with early-phase GVHD were higher than those with late-phase GVHD, suggesting that sST2 is clinically useful in predicting the early phase of GVHD. As acute GVHD often develops in the early phase of HSCT, it is advantageous to establish biomarkers that can predict the onset of GVHD at earlier time points.

A soluble form of ST2 is released from endothelial cells, epithelial cells, and fibroblasts in response to inflammatory stimuli [30,31]. A previous study has shown that sST2 levels are associated with conditioning intensity; sST2 levels were two to four times higher after the MAC regimen than the RIC regimen [4]. The present study showed that sST2 levels after MAC were relatively higher than those after RIC. To reduce the effect of conditioning intensity on sST2 levels, we also examined the predictive values of the day 14/day 0 sST2 ratio for acute GVHD, but we did not detect any relationship with the development of GVHD (data not shown). Furthermore, we examined the association between sST2 and targets of GVHD involvement (skin, gastrointestinal tract, and liver) and found that most of the patients in the high-sST2 group developed gastrointestinal GVHD, which is thought to occur due to endothelial damage. These findings imply that the release of sST2 during GVHD exacerbation partly depends on the degree of endothelial injury occurring after conditioning therapy.

On the other hand, examining the association of sST2 with other complications and mortality is also important. Vander Lugt et al. [4] showed that high sST2 levels were associated with NRM within 6 months after HSCT. Moreover, other studies showed that high sST2 levels had a similar association with increased risk of NRM [3,12,13]. In this study, although it was difficult to evaluate this issue since only four patients developed NRM, they all showed high sST2 levels on day 14. Notably, they died of sepsis, graft failure, TMA, and pneumonia, without any GVHD, and all of them developed VOD. Recent studies demonstrated that high sST2 levels were significantly associated with TMA [15] and VOD [16], both of which are characterized by endothelial cell injury. In accordance with these observations, two patients who developed TMA and five who developed VOD showed high sST2 levels on day 14, even in our small cohort. In addition, the correlation of sST2 with CRP and ferritin suggests that sST2 is released under a variety of inflammatory conditions. Several studies have shown that pro-inflammatory cytokines and their receptors are potential GVHD biomarkers, but some factors other than GVHD contribute to these cytokines’ release (e.g., TNF-α, TNFR1, IL-6) [5,6,32]. Therefore, caution should be taken when diagnosing patients who have high sST2 levels as sST2 is not a specific biomarker for acute GVHD.

### Study Limitations

The limitations of this study include the small number of patients and the heterogeneous patient populations and transplant procedures, such as conditioning regimen, donor source, and disease status. Even though we conducted detailed analyses of sST2 expression patterns in individual patients, it was difficult to exclude the multiple factors that can cause an increase in sST2 (e.g., cardiac overload or infection).

## Conclusion

We revealed that sST2 levels increased not only in patients with acute GVHD but also in those with other life-threatening complications, such as TMA, VOD, and severe infection, as identified during patient monitoring. Although these complications often overlap with each other in the clinical settings for HSCT, gastrointestinal GVHD, TMA, and VOD, all of which are linked to endothelial injury, may be key complications related to high sST2 release. The early assessment of sST2 after HSCT may be a predictive indicator for acute GVHD and other transplant-related complications. Further studies with larger sample sizes and serial monitoring are needed to clarify the clinical value of sST2.

## Figures and Tables

**Table 1:**
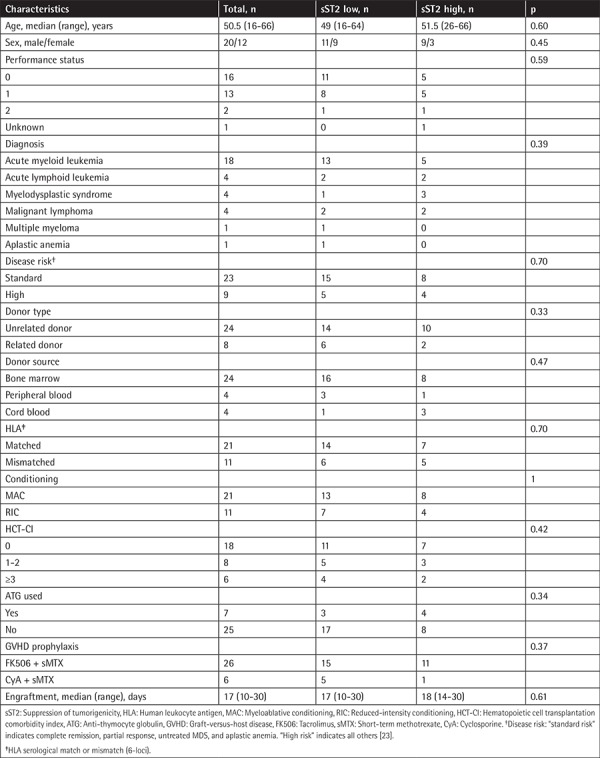
Patient characteristics.

**Table 2:**
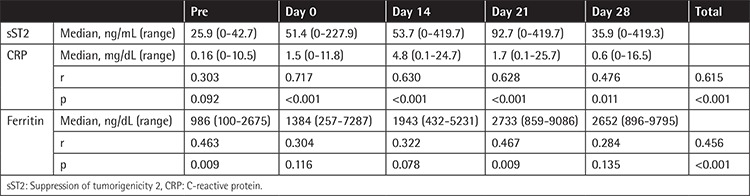
Correlation between suppression of tumorigenicity 2 and other inflammatory markers.

**Table 3:**
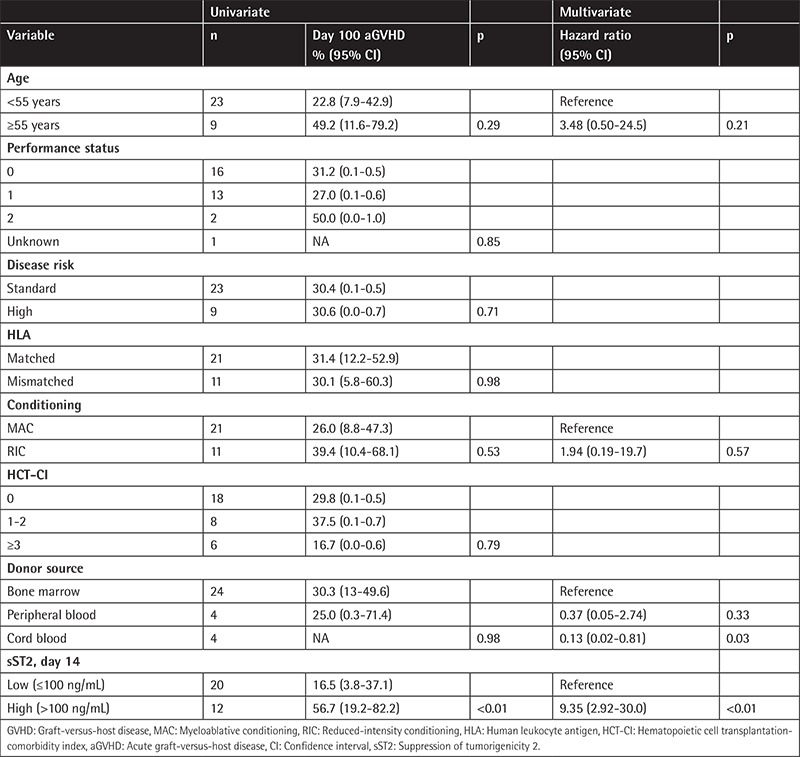
Univariate and multivariate analyses for acute graft-versus-host disease.
